# Green Preparation of S, N Co-Doped Low-Dimensional C Nanoribbon/C Dot Composites and Their Optoelectronic Response Properties in the Visible and NIR Regions

**DOI:** 10.3390/ma17174167

**Published:** 2024-08-23

**Authors:** Xingfa Ma, Xintao Zhang, Mingjun Gao, You Wang, Guang Li

**Affiliations:** 1 School of Environmental and Material Engineering, Center of Advanced Functional Materials, Yantai University, Yantai 264005, China; zhangxintao@ytu.edu.cn (X.Z.); gaomj@ytu.edu.cn (M.G.); 2 National Laboratory of Industrial Control Technology, Institute of Cyber-Systems and Control, Zhejiang University, Hangzhou 310027, China; king_wy@zju.edu.cn (Y.W.); guangli@zju.edu.cn (G.L.)

**Keywords:** polymer-derived C/C nanocomposite, interfacial charge transfer by photo-triggering, photocurrent switching, broadband light range, tentacle sensitivity

## Abstract

The green production of nanocomposites holds great potential for the development of new materials. Graphene is an important class of carbon-based materials. Despite its high carrier mobility, it has low light absorption and is a zero-bandgap material. In order to tune the bandgap and improve the light absorption, S, N co-doped low-dimensional C/C nanocomposites with polymer and graphene oxide nanoribbons (the graphene oxide nanoribbons were prepared by open zipping of carbon nanotubes in a previous study) were synthesized by one-pot carbonization through dimensional-interface and phase-interface tailoring of nanocomposites in this paper. The resulting C/C nanocomposites were coated on untreated A4 printing paper and the optoelectronic properties were investigated. The results showed that the S, N co-doped C/C nanoribbon/carbon dot hybrid exhibited enhanced photocurrent signals of the typical 650, 808, 980, and 1064 nm light sources and rapid interfacial charge transfer compared to the N-doped counterpart. These results can be attributed to the introduction of lone electron pairs of S, N elements, resulting in more transition energy and the defect passivation of carbon materials. In addition, the nanocomposite also exhibited some electrical switching response to the applied strain. The photophysical and doping mechanisms are discussed. This study provides a facile and green chemical approach to prepare hybrid materials with external stimuli response and multifunctionality. It provides some valuable information for the design of C/C functional nanocomposites through dimensional-interface and phase-interface tailoring and the interdisciplinary applications.

## 1. Introduction

The properties of nanomaterials are strongly dependent on their size and dimensional effects. These properties are rooted in the surface defects and grain boundary spacing of the material. Graphene is a bandgap-free material, whereas graphene nanoribbons have a bandgap due to quantum confinement effects of their width and edge effects. Graphene nanoribbons have been prepared in many ways, such as lithography, plasma-etching techniques, and bottom-up synthesis by polymerization of molecular precursors, and graphene oxide nanoribbons have been prepared from the open zipper of carbon nanotubes. Among these, the graphene oxide nanoribbons made from the open zipper of carbon nanotubes show more surface defects, a large length-to-diameter ratio, and good flexibility, which improves the charge transfer between grain boundaries of the nanocomposite. However, their surfaces and edges with various functional groups, such as -COOH, -OH, and epoxy, are also the basis for surface modification and functionalization of materials. These groups are not favorable for charge transport. Analysis of the relevant literature shows that, despite its high carrier mobility and broad spectral absorption, graphene is limited in light absorption (the light absorption is very low, and the optical response of graphene and graphene nanoribbons is extremely weak) [1]. To improve light harvesting for optoelectronic or interdisciplinary applications, it is often necessary to integrate other components with conventional semiconductor materials or other functional materials to exploit their excellent properties to achieve synergistic or complementary behavior [1,2,3,4,5,6,7,8,9,10,11,12,13,14,15,16,17,18,19,20,21,22,23,24,25,26,27,28,29,30,31,32,33,34,35,36,37,38,39,40,41,42,43,44,45,46,47,48,49,50,51,52,53,54,55,56,57,58,59,60,61,62,63,64,65,66,67,68,69,70,71,72,73,74,75,76,77,78,79,80,81,82,83,84,85,86,87,88,89,90,91,92,93,94,95,96,97,98,99,100,101,102,103,104,105,106,107,108,109,110,111,112,113,114,115].

Many different materials can be used to functionalize carbon materials to improve the optical response, such as metals, heavily doped oxides or sulfides with plasma properties, inorganic functional materials, organic functional small molecules, and functional polymers. The material systems involved are graphene, three-dimensional graphene composites, graphene van der Waals heterostructures [2,3,4,5,6,7,8,9,10,11], reduced graphene oxide and its nanocomposites [12,13,14], graphene nanoribbons and their composites [1,15,16,17,18,19,20,21,22,23,24,25,26,27,28,29,30,31,32,33,34], graphene quantum dots [35,36,37,38,39,40,41,42], carbon quantum dots [43], carbon nanotubes [44,45,46,47,48,49,50], graphene/C_60_ hybrids [51,52], carbon nanotube/graphene hybrids [53,54,55,56,57,58,59,60], graphene/perovskite hybrids [61,62,63,64,65,66,67], metal oxide/graphene heterostructures [68,69,70,71,72,73], various metal sulfide/graphene heterostructures [74,75,76,77,78,79,80,81,82,83,84,85,86,87], graphene/black phosphorus heterostructures, graphene/h-BN van der Waals heterostructures [88,89,90], perylene diimide/reduced graphene oxide, P3HT/graphene, hybrid graphene/metal-organic frameworks [91,92,93,94,95,96], graphene/silicon materials [97,98,99,100,101,102,103,104,105,106], hybrid graphene/GaAs, graphene/InAs, graphene on self-assembled InGaN quantum dots [107,108,109,110,111,112,113,114,115], etc.

Reviewing the relevant research progress of carbon-based nanocomposites shows that most of the research has been focused on perovskites, metal sulfides (MoS_2_, MoSe_2_, WS_2_, WSe_2_, ReS_2_, PbS, etc.), and conventional semiconductor (such as silicon materials, etc.) material systems, mainly based on the fact that these materials have good optical absorption and mature processing technology, which could improve the optical response and photoelectronic properties.

C/C dimensional composites are a kind of important material due to the good biocompatibility integrating the size and dimensional effects. In the field of carbon materials, most studies have been carried out on graphene, reduced graphene oxide, graphene nanoribbons, graphene quantum dots, carbon quantum dots, carbon nanotubes, graphene/C_60_ hybrids, carbon nanotube/graphene hybrids, etc. Among carbon materials, polymer-derived carbon materials, as a type of ordered and disordered hybrid material, represent another type of carbon nanostructure with potential applications in photoelectric and photoluminescent multifunctional composites [116,117,118]. However, due to the high number of defects in the polymer-derived carbon material, these defects can act as scattering centers that enhance light harvesting. In the field of polymers, sodium alginate (SA) is one of the biocompatible polymers. The presence of the -COOH group easily leads to polymer modification. The preparation of hybridized functional nanocomposites using biocompatible natural polymers can be considered as a green preparation technique for the development of multifunctional nanocomposites. Polymer-derived carbon materials can be easily doped with heteroatoms. Lone pairs of electrons of the doping elements can be introduced to increase the chances of n-π* jumps, which tailor the photophysical properties. Multi-element doping can also be easily achieved by polycondensation and mixing of some precursors, such as N, S element precursors. N, S elements have some lone pairs of electrons, which can change the structure of the energy level of carbon nanomaterials. They are is also widely used in the doping of non-metallic elements and energy band modulation of inorganic functional materials. The graphene oxide nanoribbons produced from carbon nanotubes have one-dimensional and two-dimensional properties. This is a one-dimensional confined process of the two-dimensional graphene. Its bandgap is strongly dependent on the width of the nanoribbons and edge effects. The one-dimensional characteristics reflect the large length-to-diameter ratio and good flexibility. The two-dimensional properties reflect the lamellar structure and unique electronic effects. The surface and edge of graphene oxide nanoribbons have some chemical groups such as -COOH, -OH, and epoxy. The edge effects include armchair-shaped edges and zigzag-shaped edges. The armchair-shaped edges and zigzag-shaped edges have a significant effect on its physical properties. The presence of these chemical groups enhances chemical and physical interactions with other components, phases, and dimensions, contributing to the construction of nanocomposites with synergistic and complementary properties, avoiding phase separation. However, the degree of oxidation of the carbon nanotubes (in the process of opening the zipper of the carbon nanotubes) would affect the charge transport of the graphene oxide nanoribbons, which would cause graphene to change from a conductor to a semiconductor and an insulator. Therefore, the combination of graphene oxide nanoribbons and polymer-derived carbon dots is expected to enhance the optical absorption and photoelectronic properties by utilizing the dimensional interface and phase interfacial interactions. Since these oxygen-containing groups tend to trap photogenerated charges, the key to material design is to balance the recombination, separation, and transfer process of photogenerated carriers. Since the interfacial contact of nanocomposites has a significant effect on the properties of the material and devices, the direct result of strong interfacial interactions of nanocomposites leads to effective interfacial charge transfer. The study of photo-triggered interfacial charge transfer not only facilitates the interdisciplinary application of materials, but also facilitates the acquisition of direct experimental evidence of interfacial interactions in nanocomposites for composite design. These studies exemplify defect engineering and interfacial engineering for the modulation of broad properties of nanocomposite or interdisciplinary applications.

Different from other carbon quantum dots or nanodots, polymer-derived carbon dots are classified as a separate class of carbon material, mainly due to their hybrid cluster structures and complex aggregation state structures. In addition, the photophysical properties of polymer-derived carbon materials are mainly focused on the visible light region. Our special interest in polymer-derived carbon materials is largely based on the diversity of polymer materials and their aggregation state structure. Due to the complex primary, secondary, and tertiary structure of polymer, the complex aggregate structure of carbon materials is inevitable in the process of polymer carbonization, and especially the combination of order and disorder. In our previous studies, the interactions between the carbon nanomaterials and metal nanodots, metal oxides, and sulfides have been investigated successively [119,120,121]. These interactions mainly focus on the component interface of nanocomposites. These interfacial studies provided a low-cost and green chemistry method to develop some advanced multifunctional materials, and have some reference for the design and application of light-responsive multifunctional and smart nanocomposites. Most of our previous research has focused on the interactions of component interfaces. The material system includes metals, oxides, sulfides, polymers, small organic molecules, and carbon materials for the design of nanocomposites with improved physical and chemical properties of materials. This study focuses on the integration of dimensional and size effects in carbon materials. The photoelectric performance can be improved by controlling the dimensional and phase interfaces of carbon nanocomposites and doping effects in the process of nanocomposite preparation in situ. This is a simple and general approach to developing a range of multifunctional low-dimensional C/C hybrid materials using abundant polymer precursors.

## 2. Materials and Methods

### 2.1. Materials

Carbon nanotubes, L-MWNT-60100, Shenzhen NanoPort Co., Shenzhen, China.

Potassium permanganate (AR), Yantai Sanhe Chemical Reagent Co., Ltd., Yantai, China.

Concentrated sulfuric acid (AR), Laiyang Fine Chemical factory, Laiyang, China.

Concentrated nitric acid (AR), Sinopharm Chemical Reagent Co., Ltd., Shanghai, China.

Sodium alginate (AR) (the purity 99.9%), Tianjin Basf Chemical Co., Ltd., Tianjin, China.

Urea (AR) (purity 99.0%), Tianjin Huihang Chemical Reagent Co., Ltd., Tianjin, China.

Thiourea (AR) (purity 99.0%), Tianjin Taixing Chemical reagent factory, Tianjin, China.

### 2.2. Preparation of Sodium Alginate Solution

Quantities of 1 g of sodium alginate and 80 mL of H_2_O were added to a 200 mL glass flask, stirred, and sonicated for 5–10 min. The sodium alginate solution was saved for future use.

### 2.3. Preparation of Graphene Oxide Nanoribbons

The graphene oxide nanoribbons used in this study are shown in our previous report [122,123]. The concentration of graphene oxide nanoribbons in this study was approximately 4.3 mg/mL.

### 2.4. Preparation of Low-Dimensional Carbon/Carbon Nanocomposite Derived from Polymer

Quantities of 40 mL sodium alginate solution and 20 mL graphene oxide nanoribbons were added and stirred for 1–3 min. A quantity of 0.5 g of thiourea was added as the sulfur source. The hydrothermal reaction conditions and sample treatment were the same as in our previous report [120] to obtain the S, N co-doped nanocomposite.

Similarly, 40 mL sodium alginate solution and 20 mL graphene oxide nanoribbons were added and stirred for 1–3 min, and 0.5 g of urea was added. The hydrothermal reaction conditions and sample treatment were the same as in our previous report [120] for comparison of the N-doped sample.

### 2.5. Characterization via SEM, TEM, UV-Vis-NIR, XRD, EDS, and Raman Spectra

The characterization via SEM (scanning electron microscope), TEM (transmission electron microscope), UV-Vis-NIR (UV-VIS-NIR spectrophotometer) and XRD (X-ray powder diffraction) was shown in our previous report [119,120]. The instruments used were ZEISS Gemini SEM500 (Germany), JEM-1011 (Japan Electronics Co., Ltd.), TU-1810 spectrophotometer (Beijing Puxi General Instrument Co., Ltd., Beijing, China), and XRD-7000 from SHIMADZU (Shimadzu, Kyoto, Japan). Energy dispersive spectroscopy (EDS) measurements were performed using a Hitachi S-4800 (HITACHI, Tokyo, Japan).

The Raman spectra were characterized as follows: the sample suspension was dip-coated onto the glass carrier sheet and dried at room temperature. The Raman spectra were determined using a PHS-3C confocal Raman spectrometer (HORIBA, Kyoto, Japan). The operating wavelength and power density of the laser radiation were 785 nm and 5 mW, respectively.

### 2.6. Photocurrent Signal of Low-Dimensional Carbon/Carbon Nanocomposite in the Visible Light and NIR

**Scheme 1:**
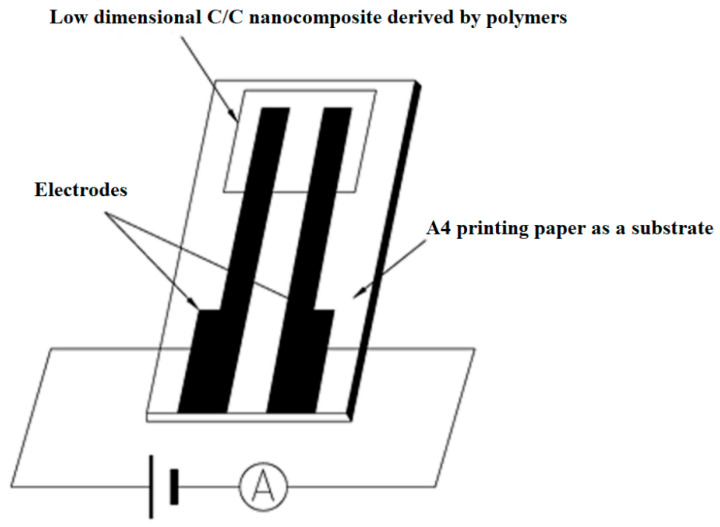
The structure of electrodes in this study for examining the photocurrent signal and tentacle sensitivity.

The determination of the photocurrent signal of the low-dimensional C/C nanocomposite in the visible light region and NIR was shown in our previous report [119]. In this study, untreated A4 printing paper was used as the substrate (Au gap electrodes on PET film in previous studies). The electrode structure is shown in Scheme 1. The photocurrent signal in some typical light sources, such as 650 nm (50, 5 mW), and 808, 980, and 1064 nm NIR (10, 20, 50, 100, and 200 mW), was determined using an LK2000A Electrochemical Work Station (LANLIKE Chemistry and Electron High Technology Co., Ltd. (Tianjin, China)) with 1 V DC bias applied, and the current of the thick film was measured by computer recording before and after irradiation of the light sources.

### 2.7. Examination of the Tentacle Sensitivity of the Low-Dimensional C/C Nanocomposite to the Applied Force

To examine the tentacle sensitivity of the low-dimensional C/C nanocomposite to the applied force, the structure of Scheme 1 was bonded to the EVA (ethyl vinyl acetate) foam (thickness approximately 2 mm) with a pressure-sensitive adhesive. The force was applied by the deformation of the EVA foam. The electrical responses were preliminarily measured using the LK2000A Electrochemical Work Station from LANLIKE Chemistry and Electron High Technology Co., Ltd. (China) under different compression forces (such as 20, 50, and 100 g weight of the balance) or finger touch with 1 V DC bias applied [124]. The current of the thick film was measured by computer recording before and after the applications of compressive forces or finger touch.

## 3. Results and Discussion

**Figure 1:**
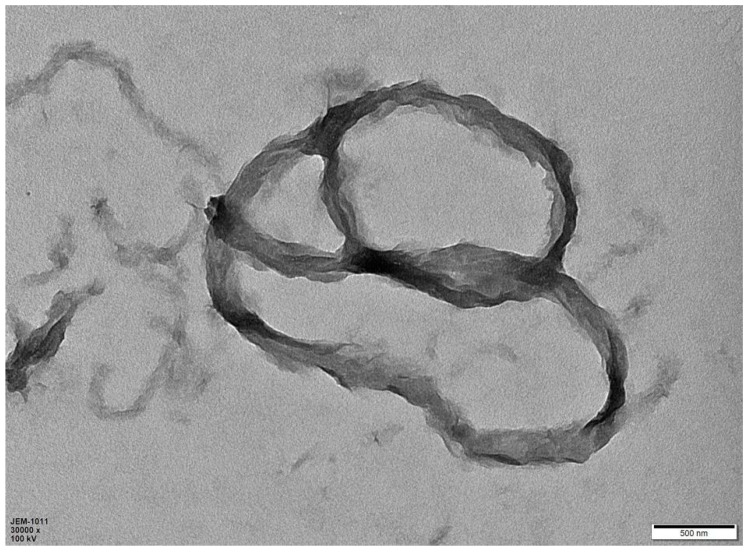
The representative TEM image of the graphene oxide ribbons used in this study (the magnification is 30,000×).

The synthesis of graphene ribbons has many methods, including lithography, plasma-etching techniques, bottom-up synthesis by polymerization of molecular precursors, and the opened zipper method of carbon nanotubes. The opened zipper method of carbon nanotubes is a simple approach to obtain graphene oxide ribbons. Due to good flexibility and some chemical groups, strong interfacial interaction with some inorganic oxide nanomaterials improves their physical and chemical properties. The entanglement of graphene oxide nanoribbons with low-dimensional oxide materials was carried out to improve the properties of nanocomposites [122,123]. The representative TEM images of the graphene oxide ribbons used in this study are shown in Figure 1.

**Figure 2:**
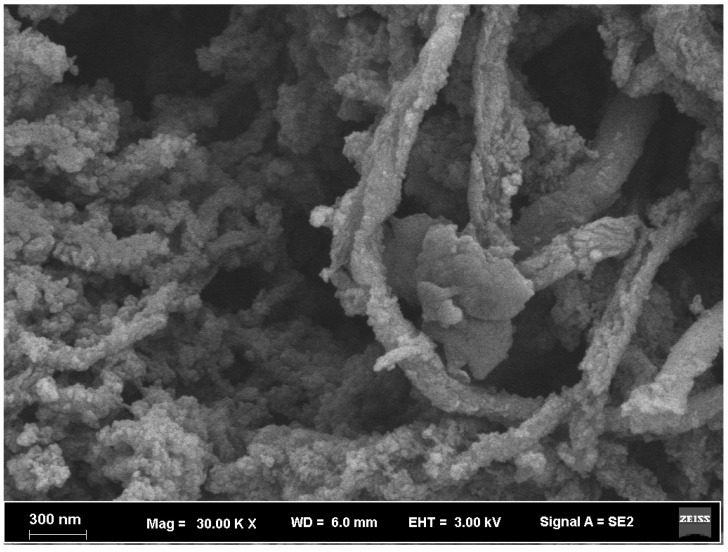
The representative SEM image of S, N co-doped low-dimensional C/C nanocomposite derived by polymer (the magnification is 30,000×).

As shown in Figure 1 of the TEM, it has a ribbon-like shape, the length is several µm, and the width is tens of nanometers. The thickness of the ribbons is very thin. The tubular structure of the carbon nanotube no longer exists. There are some fragments of the nanoribbons. Most of the graphene oxide nanoribbons showed good flexibility because they were in a wavy state. Such a long ribbon-like structure shows more edges and edge defects, including armchair-shaped edges and zigzag-shaped edges. In the presence of graphene oxide nanoribbons, S, N co-doped C/C nanocomposites were prepared by polymer hydrothermal carbonization. The representative SEM image of S, N co-doped nanocomposites obtained by polymer carbonization in the presence of graphene oxide ribbons is shown in Figure 2.

**Scheme 2:**
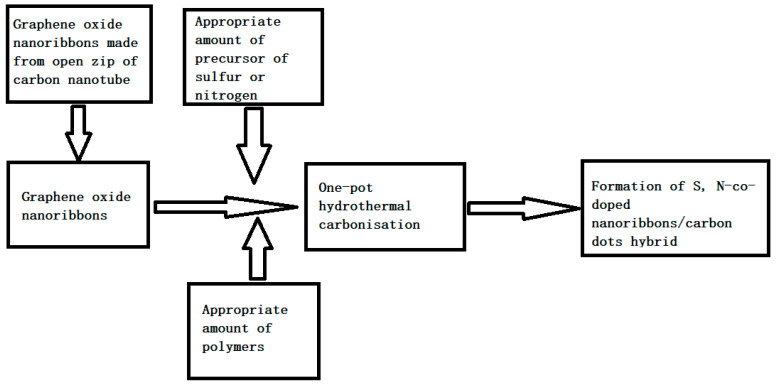
The formation process of S, N co-doped low-dimensional C/C nanocomposite derived by polymer.

As shown in Figure 2 of the SEM, some nanoparticles were attached to the surface of the graphene oxide ribbons, the size of which is several to tens of nanometers. These nanoparticles should belong to the polymer-derived carbon dots. Since polymer-derived carbon materials have more defects, these defects can be used as scattering centers to improve the light-harvesting ability. The graphene oxide nanoribbons have a high proportion of C sp^2^ hybridization, which is conducive to the transport of photogenerated carriers. Graphene oxide nanoribbons contain a large number of oxygen-containing groups, which are not favorable for charge transport. However, the hydrothermal treatment process could remove some of the oxygen and convert it into reduced graphene oxide nanoribbons, which will improve the charge transport performance. Therefore, the nanocomposites prepared in this study are the integration of dimensions of carbon nanomaterials and the combination of the order and disorder of carbon, including the dimensional interface and phase interface. Improving their photogenic charge extraction ability is a major challenge of interfacial interaction and defect engineering. Since the preparation of the material is very simple, the formation process of low-dimensional C/C nanocomposite derived from polymer is shown in Scheme 2.

**Figure 3:**
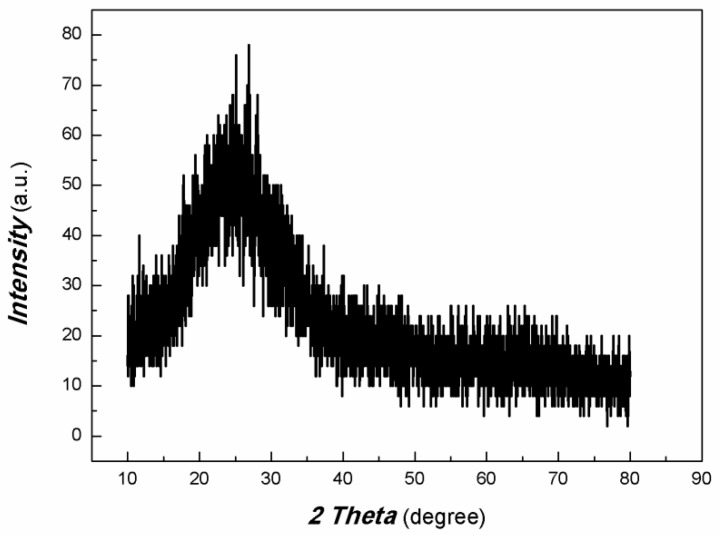
The XRD results of the S, N co-doped C/C nanocomposite derived by polymer.

The XRD results of the S, N co-doped polymer-derived low-dimensional C/C nanocomposite are shown in Figure 3.

As shown in Figure 3, the amorphous steamed bun peaks of the material are very prominent. This indicates that the disorder content of the carbon material is quite high. These disordered carbons are mainly derived from polymers. The diffraction peaks at 26.3° is the peak of the (002) plane of graphite-2H (PDF# 41-1487) for the polymer-derived low-dimensional C/C composite. This should be due to the addition of some graphene oxide nanoribbons. As the amount of graphene oxide nanoribbons added is very small, the diffraction peak is not strong.

**Figure 4:**
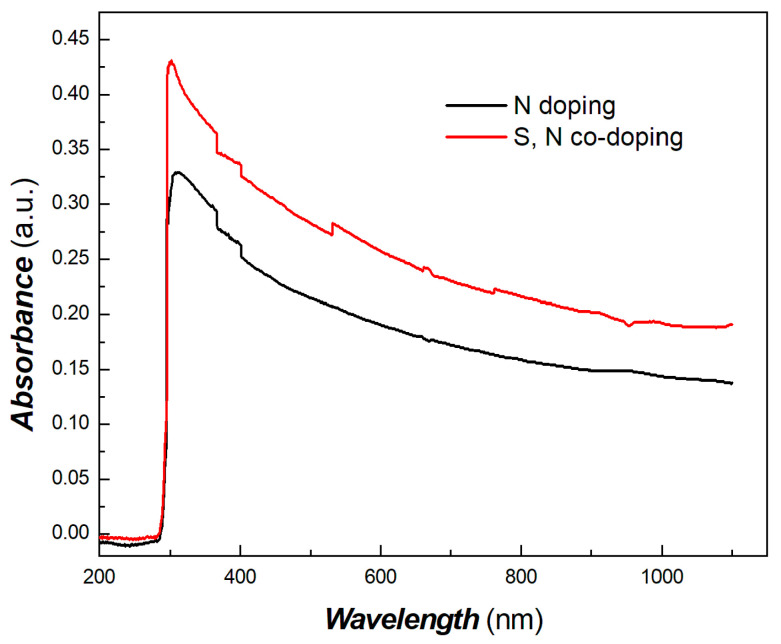
The UV−Vis−NIR absorbance curve of C/C nanocomposite derived by polymer.

The UV-Vis-NIR absorbance curve of S, N co-doped low-dimensional C/C nanocomposite derived from polymer is shown in Figure 4.

As shown in Figure 4, the low-dimensional C/C nanocomposite derived from the polymer was found to have similar absorption in the visible and NIR regions. N doping and N, S co-doping have little effect on the light absorption property. The light absorption of the C/C nanocomposite comes from the graphene oxide nanoribbons having a bandgap due to quantum confinement effects of their width and edge effects. The edge effects include armchair-shaped edges and zigzag-shaped edges. On the other hand, polymer-derived carbon has a large number of defects that act as scattering centers to improve light harvesting. The optical absorption of polymer-derived carbon nanocomposite is a comprehensive result of multi-element doping, which is worth discussing.

**Figure 5:**
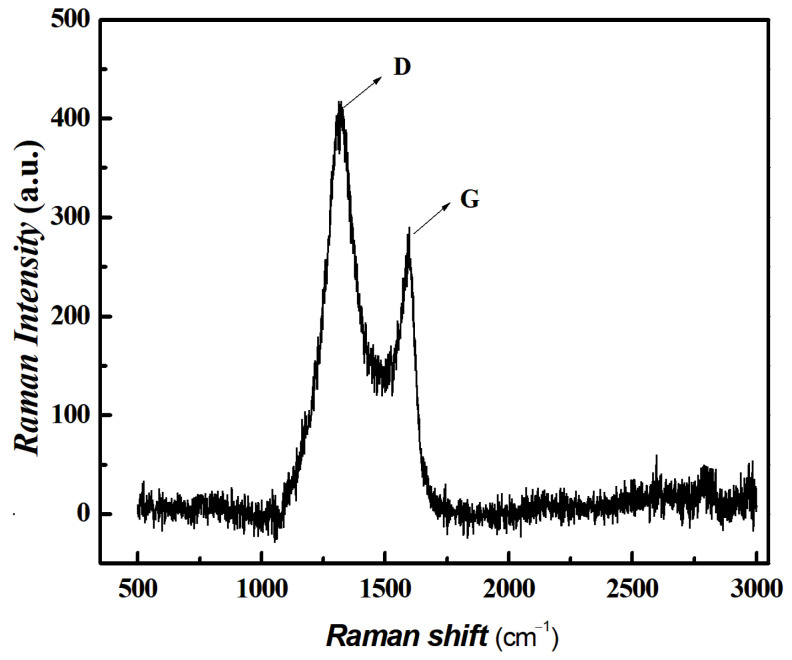
The Raman spectra of low-dimensional C/C nanocomposite derived by polymer.

The Raman spectra of the S, N co-doped polymer-derived low-dimensional C/C nanocomposite are shown in Figure 5.

In the experiment of studying the Raman spectra of S, N co-doped C/C nanocomposite derived by polymer, it was found that the resulting low-dimensional C/C nanocomposite had some fluorescence when using the 532 nm and 633 nm wavelength laser resource excitation. Since the probability of Raman scattering is very low, it is difficult to characterize the Raman signal of fluorescent materials. When the excitation was performed using a 785 nm light source, the Raman signal could be obtained. It is shown in Figure 5.

As shown in Figure 5, the band at about 1270 cm^−1^ belongs to the D peak of the disordered structure, which is located in the wide range of 1107–1452 cm^−1^, and the peak value of the D band is much higher than that of the G band. This shows that the defects of the material are very rich and there are many kinds of disordered phase. The band at about 1580 cm^−1^, corresponding to C sp^2^ of the G peak, is also very strong in the sample of the polymer-derived C/C nanocomposite, and is also located in the broad range of 1516–1660 cm^−1^. It shows that the ordered structure of the material is also varied, i.e., short-range order, medium-range order, long-range order, ordered structures containing some disorder phases, disordered structures having some short-range order, etc. Such complex structures mainly come from the evolution of polymers through the carbonization process. Part of the ordered structure comes from the addition of graphene oxide nanoribbons.

Otherwise, in the S, N doping experiments, some amount of thiourea and urea was added. Urea is very similar in structure to thiourea. Urea has NH_2_ groups and the O element, and thiourea contains NH_2_ groups and the S element. The NH_2_ groups of thiourea and urea can be cross-linked with -COOH groups of polymer. The degree of crosslinking of the polymers affects the photophysical properties of the polymer-derived carbon materials. The addition of thiourea results in S, N co-doping. The lone electron pair of the S, N atom participates in the electron transition process and increases some transition energy, which is favorable for photoexcited transitions. At the same time, the lone electron pairs of S, N can also passivate the defects of carbon materials.

Although there are many mechanisms of light detection, such as photoconductance (PC), photovoltaic, photothermoelectric (PTE), photogating (PG), and bolometric effects, these mechanisms depend not only on the material properties, but also on the device structure. The photoelectronic properties are mainly based on the following mechanisms: (1) generation of electron–hole (e-h) pairs by absorption of photons of different wavelengths, and (2) separation of carriers to generate a junction current and/or junction voltage. The study of photoelectric properties of materials not only includes light detection and solar cell fields, but also has a wide range of applications in interdisciplinary fields such as light synthesis, photocatalysis, imaging, and biomedicine. The generation, separation, and transfer of photogenic charges involve many material factors, such as the bandgap width, interfacial interactions, defect engineering, formation of built-in electric field, and applied bias voltage. Therefore, the study of light-triggered charge transfer behavior has attracted interdisciplinary attention. In our previous studies [119], most of our investigations used a PET film substrate and Au gap electrodes to study the photoelectric signal. However, the photoelectric signals could be obtained using untreated A4 printing paper as a substrate due to its low cost for part of the nanocomposite system. Several typical light sources, such as 650, 808, 980, and 1064 nm, were chosen to study the photoelectric signals from the visible to the NIR region.

**Figure 6:**
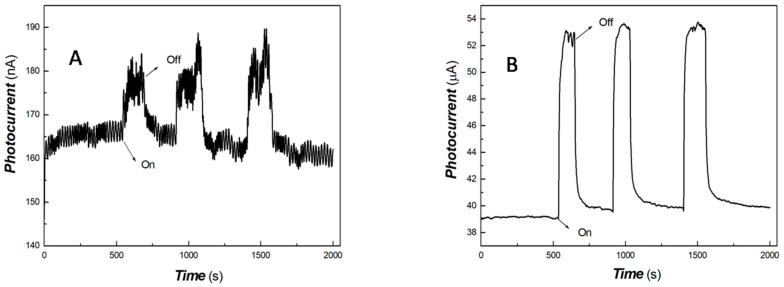
The transient-state photocurrent responses of C/C nanocomposite derived by polymer for 50 mW 650 nm ((**A**) N doping; (**B**) S, N co-doping).

**Figure 7:**
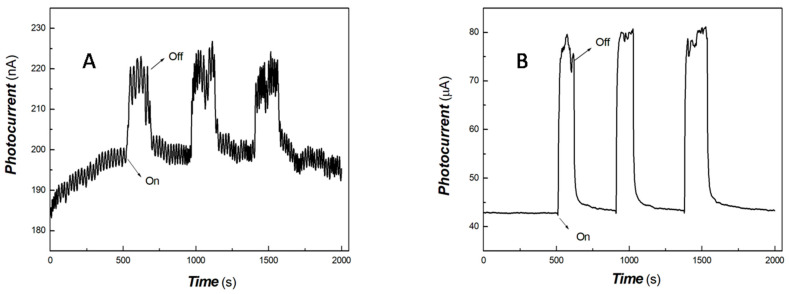
The transient-state photocurrent responses of C/C nanocomposite derived by polymer for 200 mW 808 nm ((**A**) N doping; (**B**) S, N co-doping).

**Figure 8:**
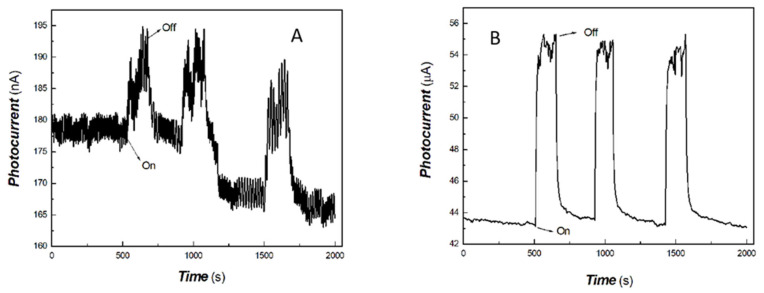
The transient-state photocurrent responses of C/C nanocomposite derived by polymer for 100 mW 980 nm ((**A**) N doping; (**B**) S, N co-doping).

**Figure 9:**
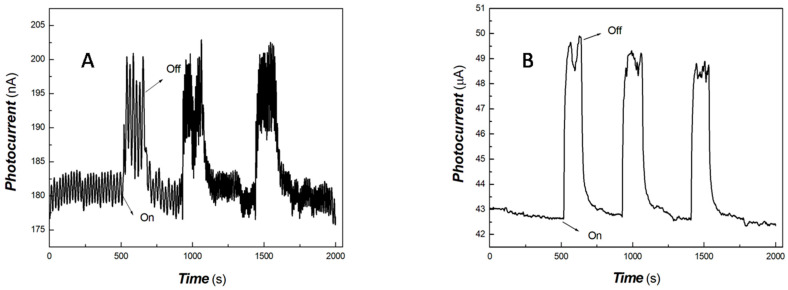
The transient-state photocurrent responses of C/C nanocomposite derived by polymer for 20 mW 1064 nm ((**A**) N doping; (**B**) S, N co-doping).

The representative photoelectric signal comparisons are shown in Figure 6, Figure 7, Figure 8 and Figure 9.

As shown in Figure 6, Figure 7, Figure 8 and Figure 9, it was found that compared to the N-doped sample, the S, N-co-doped low-dimensional polymer-derived C/C nanocomposite exhibits enhanced photocurrent signals for 50 mW 650 nm, 200 mW 808 nm, 100 mW 980 nm, and 20 mW 1064 nm light sources. Not only is the baseline current increased by 2–3 orders of magnitude (from nA to μA orders), but the on/off ratio is also significantly improved. The on/off ratio, response time, and recovery time are summarized in Table 1.

**Scheme 3:**
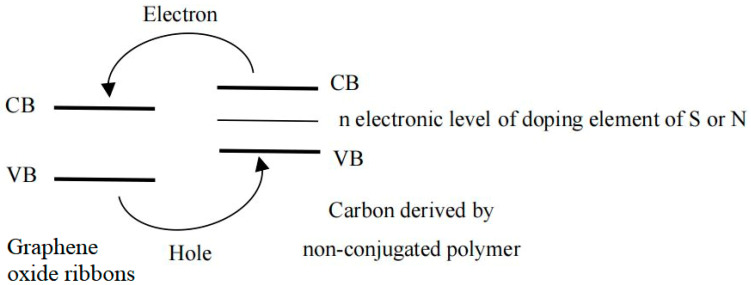
The light-triggered charge transfer process of S, N co-doped low-dimensional C/C nanocomposite derived by polymer.

As shown in Table 1, the on/off ratio of the S, N-co-doped low-dimensional polymer-derived C/C nanocomposite is significantly higher than that of the N-doped counterpart. Under the premise that the on/off ratio is equal, the response speed is obviously improved for the S, N-co-doped low-dimensional polymer-derived C/C nanocomposite. Overall, the S, N-co-doped low-dimensional polymer-derived C/C nanocomposite is more sensitive to the near-infrared region. This is mainly due to the passivation of the defect by the electronic effects of the S, N atom, which improves the ability of photogenerated carriers to be extracted. The discussed mechanism is shown in Scheme 3.

As shown in Scheme 3, the S, N element co-doping not only increases the transition level by light excitation, but also effectively passivates the defects of carbon materials, and the interfacial charge transfer by light triggering is enhanced. The broadband photocurrent signals are mainly due to the defect engineering and bandgap tailoring of carbon materials.

In order to further explore the doping mechanism, comparative EDS was carried out. The results are shown in Table 2.

As shown in Table 2, it was found that the main elemental contribution of the S-, N-co-doped and N-doped low-dimensional C/C nanocomposites is the C, O, N, and S elements. A small number of other elements, such as Na, Mg, Al, Si, P, Cl, and Ca, may come from the polymer precursor due to sodium alginate being produced with seaweed. The big difference in content is the S and N elements. Although they all have lone pairs of electrons, the sulfur atom has more lone pairs of electrons than the N atom, and its atomic radius is also larger than that of the N atom. Therefore, the overlap of the sulfur atom and carbon material electron cloud is larger than that of the N atom, and there are more opportunities for electron transition excitation, which is favorable for the extraction of photogenic charge. Based on atomic percentages, the sulfur content of S, N-doped low-dimensional C/C nanocomposites is 125 times higher than that of N-doped low-dimensional C/C nanocomposites. The nitrogen content of N-doped low-dimensional C/C nanocomposites is 1.2 times higher than that of S, N-co-doped low-dimensional C/C nanocomposites. Therefore, it is expected that sulfur doping has a more significant effect on their photophysical performance. Furthermore, the atomic ratio of the main doping element (such as O, N, and S) to the carbon element in the C/C nanocomposite was analyzed. The results showed that the atomic ratio of the main doping element (such as O, N, and S) to the carbon element in the S, N-co-doped low-dimensional C/C nanocomposites (about 78.43%) is much higher than that of the N-doped sample (about 67.04%). Therefore, multi-element doping in polymer-derived carbon materials is easier to achieve. The enhancement of photogenerated carrier extraction ability may be related to the synergistic effect of multi-element doping. This needs to be further investigated.

**Figure 10:**
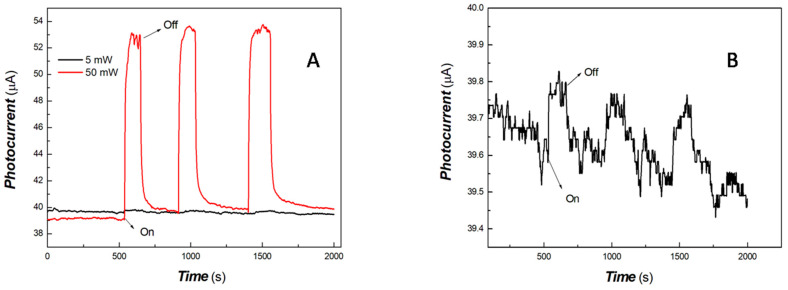
The dependence of photocurrent responses of S, N co-doped low-dimensional C/C nanocomposite derived by polymer on the power of excitation to 650 nm ((**A**) 50, and 5 mW; (**B**) 5 mW local amplification).

**Figure 11:**
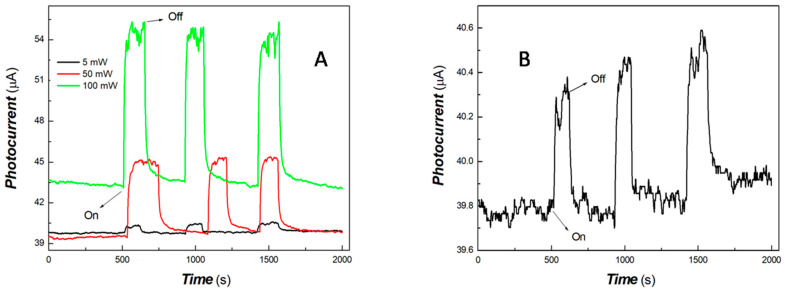
The dependence of photocurrent responses of S, N co-doped low-dimensional C/C nanocomposite derived by polymer on the power of excitation to 980 nm ((**A**) 100, 50, and 5 mW; (**B**) 5 mW local amplification).

Based on the above research results, the dependence of the photocurrent responses of S, N co-doped low-dimensional polymer-derived C/C nanocomposites on the power of typical excitation light sources was investigated. The results are shown in Figure 10 and Figure 11.

As shown in Figure 10 and Figure 11, it was found that the S, N co-doped low-dimensional polymer-derived C/C nanocomposite still shows good photocurrent for up to 5 mW 650 and 980 nm light sources. The local amplification is shown in Figure 10B and Figure 11B. The photosensitivity to 980 nm is significantly better than that of 650 nm, indicating greater near-infrared sensitivity for the resulting C/C nanocomposite.

It can be seen from the above results that the S, N co-doped C/C nanocomposite in this study showed good photocurrent responses from the visible region to part of the NIR. It also shows that this method for enhancing optical absorption and photocurrent extraction is feasible. Although the field of C/C composites is wide, including structural composites and functional composites, this is a simple, low-cost way to develop C/C functional nanocomposites.

**Figure 12:**
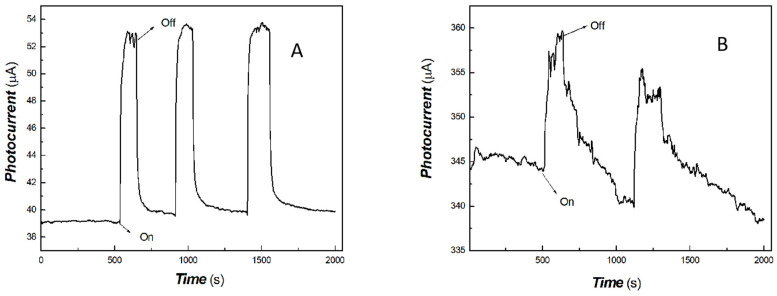
The transient-state photocurrent comparative responses of C/C nanocomposite derived by polymer to 50 mW 650 nm ((**A**) S, N co-doped reduced graphene oxide nanoribbons/carbon dots derived from sodium alginate; (**B**) reduced graphene oxide/carbon materials derived from starch).

**Figure 13:**
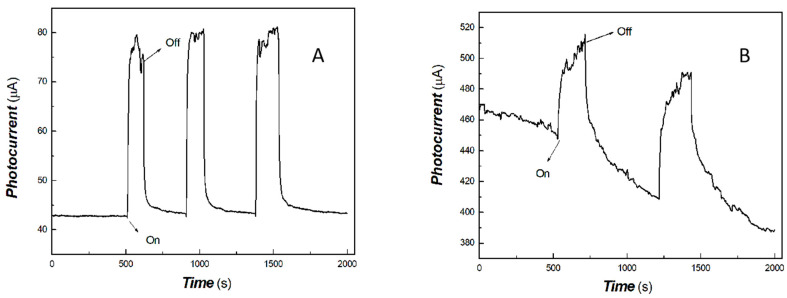
The transient-state photocurrent comparative responses of C/C nanocomposite derived by polymer to 200 mW 808 nm ((**A**) S, N co-doped reduced graphene oxide nanoribbons/carbon dots derived from sodium alginate; (**B**) reduced graphene oxide/carbon materials derived from starch).

**Figure 14:**
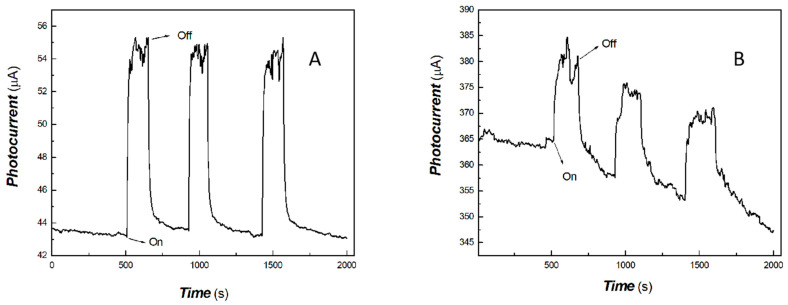
The transient-state photocurrent comparative responses of C/C nanocomposite derived by polymer to 100 mW 980 nm ((**A**) S, N co-doped reduced graphene oxide nanoribbons/carbon dots derived from sodium alginate; (**B**) reduced graphene oxide/carbon materials derived from starch).

**Figure 15:**
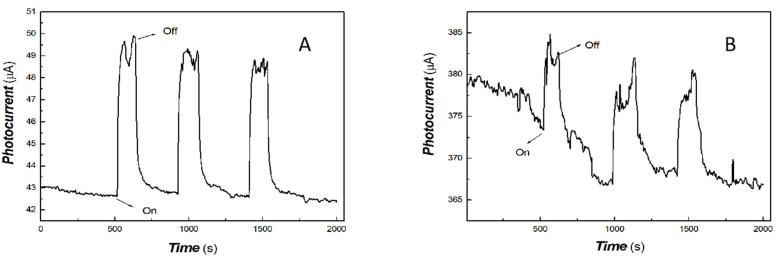
The transient-state photocurrent comparative responses of C/C nanocomposite derived by polymer to 20 mW 1064 nm ((**A**) S, N co-doped reduced graphene oxide nanoribbons/carbon dots derived from sodium alginate; (**B**) reduced graphene oxide/carbon materials derived from starch).

This method is also used in the synthesis of several other low-dimensional C/C hybrid functional materials from a variety of polymers, such as reduced graphene oxide/carbon derived from starch, or various other polymeric materials with good biocompatibility in our study. Some similar results have also been obtained. Some representative results were selected for comparison. The results are shown in Figure 12, Figure 13, Figure 14 and Figure 15. This provides a general and green chemistry method for the synthesis of a range of functional hybrid materials. It also expands some applications of these multifunctional nanocomposites and devices.

As shown in Figure 12B, Figure 13B, Figure 14B and Figure 15B, although the starch-derived reduced graphene oxide/carbon materials exhibit some photoelectric properties in the broadband light region (from the visible region to the NIR), their response speed and photocurrent sensitivities are still much lower than those of the S, N co-doped polymer-derived low-dimensional C/C nanocomposite (they are shown in Figure 12A, Figure 13A, Figure 14A and Figure 15A). This shows that different polymer systems and S, N co-doping have significant effects on the photoelectric properties, which need to be further investigated. This also shows the complexity of the aggregated structures and cyclization and carbonization processes of different polymers on the ordered and disordered carbon produced.

**Figure 16:**
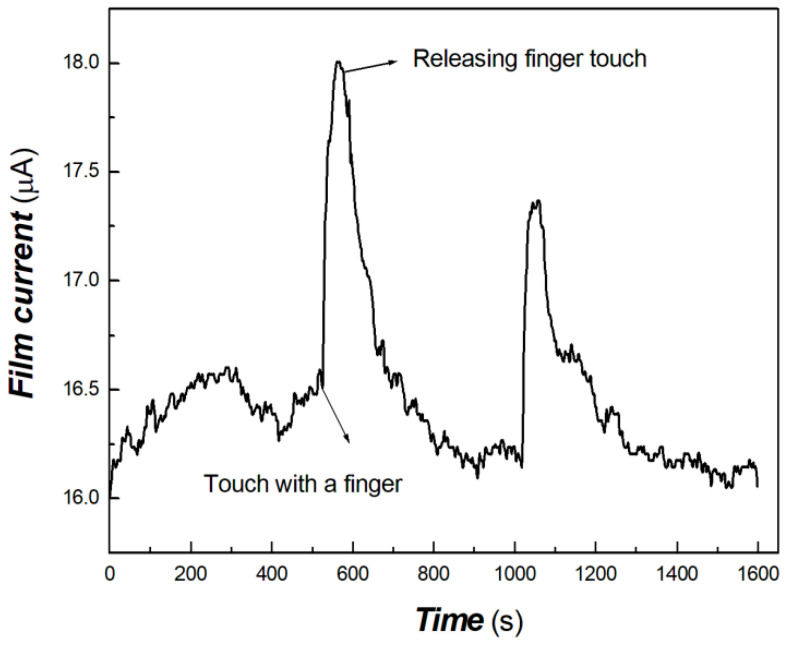
The tentacle sensitivity of S, N co-doped low-dimensional C/C nanocomposite derived by polymer to the compression force of a finger touch.

In order to investigate the stimulus response of materials, the tentacle sensitivity of the S, N co-doped low-dimensional C/C nanocomposite to force was preliminarily investigated. The prototype device for investigating the photocurrent behavior was subjected to a finger-touch force. The electrical response is shown in Figure 16.

**Figure 17:**
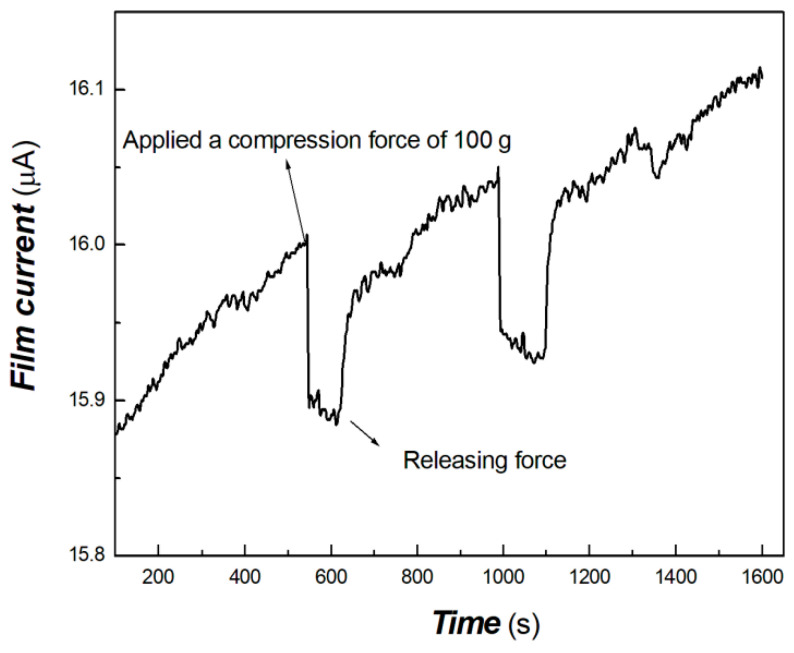
The tentacle sensitivity of S, N co-doped low-dimensional C/C nanocomposite derived by polymer to compression force of about 100 g.

As shown in Figure 16, the film current increases when the compressive force is applied with a finger touch. Conversely, the current decreases when the compressive force is released. This shows that the S, N co-doped low-dimensional C/C nanocomposite exhibits some force sensitivity. Since it is not easy to control the magnitude of the force by touch, a 100 g weight was applied to the prototype device and the electrical response is shown in Figure 17.

As shown in Figure 17, the film current decreases when the compression force is applied with a 100 g weight. Conversely, the current increases when the compression force is released. Since the S, N co-doped low-dimensional C/C nanomaterial was coated on the A4 printing paper, the paper is a kind of natural polymer. Applying the appropriate compression force would cause the distance between the nanoparticles to increase and the film current to decrease. If a larger compression force were applied (such as the touch of a finger), the distance between the nanoparticles would decrease further and the film current would increase.

**Figure 18:**
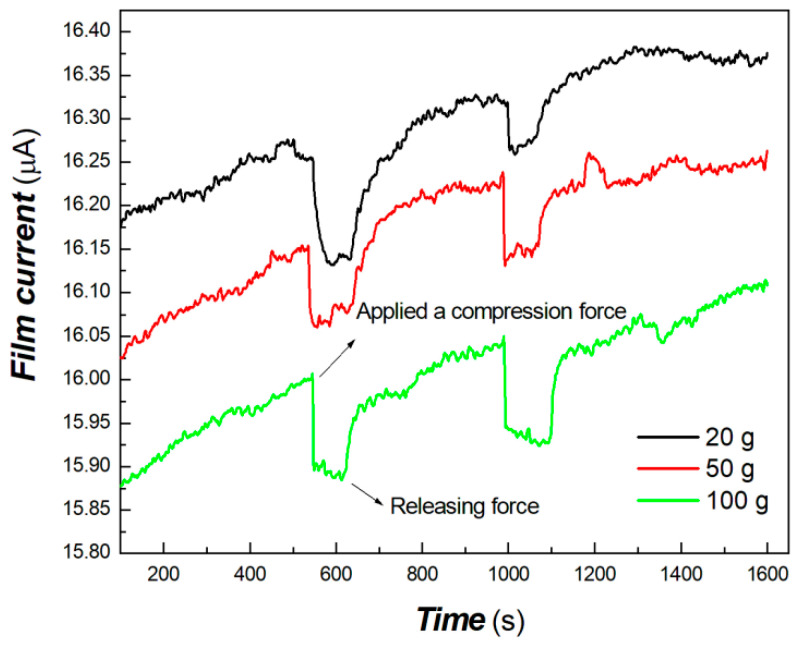
The effects of different external forces applied (20, 50, and 100 g) on the tentacle sensitivity of S, N co-doped low-dimensional C/C nanocomposite derived by polymer (the external force applied is 100 g, 50 g, and 20 g).

The effects of different weights on the sensitivity of the film were also examined. The results are shown in Figure 18.

As shown in Figure 18, its sensitivity was highly dependent on the magnitude of the compressive force. Since the force is applied by deforming the polymer, EVA foam is a viscoelastic material. Hysteresis is inevitable. The improvement of hysteresis can be achieved by changing the polymer used, and further research is needed. This is only a preliminary study of the material’s response to force.

In conclusion, this study provides a simple and general approach to prepare the multifunctional S, N co-doped low-dimensional C/C nanocomposite with enhanced photophysical properties for interdisciplinary applications. It also demonstrates the simplicity and effectiveness of multi-element doping of polymer-derived carbon materials. The scope and applications of C/C composites have been expanded.

## 4. Conclusions

In conclusion, S, N co-doped low-dimensional C/C nanocomposites were obtained by one-pot carbonization with polymer. The results showed that the S, N co-doped polymer-derived C/C nanocomposite coated on untreated A4 printing paper exhibited enhanced broadband spectral photocurrent signals compared to the N-doped sample. This has potential applications in broadband flexible photodetectors, tentacle sensors, or interdisciplinary areas for light harvesting. Dimensional integration and phase–phase–interface interaction are important issues in the design of nanocomposites. Doping with S, N not only increases the transition level, but also effectively passivates the defects of carbon materials, improving the interfacial photogenerated charge transfer. The photophysical and doping mechanisms are discussed. This study provided a green chemical and general method for the synthesis of low-dimensional C/C nanocomposites, which exhibited good photocurrent signals in the visible and NIR regions. It also helps in the design of light-responsive low-dimensional C/C hybrids using polymer systems and by tailoring dimensional and phase interfaces. The variety and application of C/C composites have been expanded.

## Figures and Tables

**Table 1:** The comparative response time, recovery time, and on/off ratio of S, N-co-doped low-dimensional C/C nanocomposites.

Excitation Light Wavelength (nm)	Sample No.	Response Time (s)	Recovery Time (s)	Ratio of On/Off
50 mW 650 nm	1# S, N-co-doped low-dimensional C nanoribbons/C nanocomposites	18.00	51.43	1.35
50 mW 650 nm	2# N-co-doped low-dimensional C nanoribbons/C nanocomposites	13.71	34.28	1.11
200 mW 808 nm	1# S, N-co-doped low-dimensional C nanoribbons/C nanocomposites	8.58	42.85	1.79
200 mW 808 nm	2# N-co-doped low-dimensional C nanoribbons/C nanocomposites	25.72	21.43	1.10
100 mW 980 nm	1# S, N-co-doped low-dimensional C nanoribbons/C nanocomposites	17.14	30.85	1.25
100 mW 980 nm	2# N-co-doped low-dimensional C nanoribbons/C nanocomposites	21.43	80.47	1.07
20 mW 1064 nm	1# S, N-co-doped low-dimensional C nanoribbons/C nanocomposites	22.28	51.43	1.15
20 mW 1064 nm	2# N-co-doped low-dimensional C nanoribbons/C nanocomposites	17.14	26.13	1.09

**Table 2:** The comparative composition of S, N-co-doped and N-doped low-dimensional C/C nanocomposites by EDS data.

**Sample No.**	**Atomic Percent (%)**	**C Element**	**O Element**	**N Element**	**S Element**	**Other Elements**
1# S, N-co-doped low-dimensional C nanoribbons/C nanocomposites		53.72	27.77	8.06	6.30	Na: 0.34; Al: 0.14; Si: 0.46; Cl: 0.14; K: 0.59; Ca: 2.47
2# N-co-doped low-dimensional C nanoribbons/C nanocomposites		59.28	18.00	21.69	0.05	Na: 0.07; Mg: 0.06; Al: 0.08; Si: 0.34; P: 0.05; Cl: 0.04; Ca: 0.34
**Sample No.**	**Weight Percent (%)**	**C Element**	**O Element**	**N Element**	**S Element**	**Other Elements**
1# S, N-co-doped low-dimensional C nanoribbons/C nanocomposites		41.46	28.55	7.25	12.98	Na: 0.50; Al: 0.24; Si: 0.83; Cl: 0.33; K: 1.48; Ca: 6.37
2# N-co-doped low-dimensional C nanoribbons/C nanocomposites		53.27	21.54	22.73	0.11	Na: 0.11; Mg: 0.11; Al: 0.19; Si: 0.72; P: 0.13; Cl: 0.09; Ca: 1.01
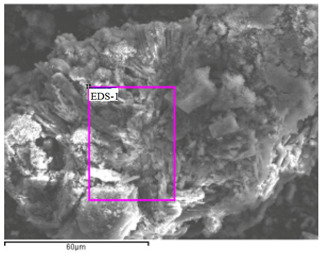			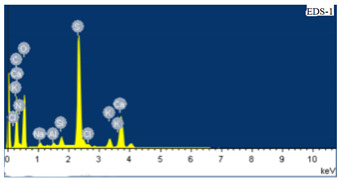			
(**a**)			(**b**)			
**1#-S, N co-doped low-dimensional C/C nanocomposite**						
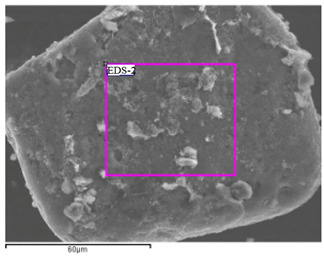			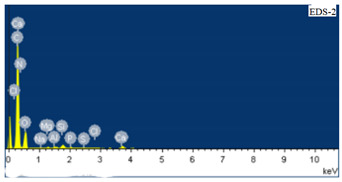			
(**a**)			(**b**)			
**2#-N-doped low-dimensional C/C nanocomposite**						

## Data Availability

The data presented in this study are available on request from the corresponding author. The data are not publicly available due to privacy.
